# High-Throughput Transcriptomic Analysis of Circadian Rhythm of Chlorophyll Metabolism under Different Photoperiods in Tea Plants

**DOI:** 10.3390/ijms25179270

**Published:** 2024-08-27

**Authors:** Zhi-Hang Hu, Meng-Zhen Sun, Kai-Xin Yang, Nan Zhang, Chen Chen, Jia-Wen Xiong, Ni Yang, Yi Chen, Hui Liu, Xing-Hui Li, Xuan Chen, Ai-Sheng Xiong, Jing Zhuang

**Affiliations:** 1 Tea Science Research Institute, College of Horticulture, Nanjing Agricultural University, Nanjing 210095, China; 2022204007@stu.njau.edu.cn (Z.-H.H.); 2023104080@stu.njau.edu.cn (M.-Z.S.); 2023804322@stu.njau.edu.cn (K.-X.Y.); 2021204040@stu.njau.edu.cn (N.Y.); 2019204042@stu.njau.edu.cn (Y.C.); lxh@njau.edu.cn (X.-H.L.); chenxuan@njau.edu.cn (X.C.); 2 State Key Laboratory of Crop Genetics & Germplasm Enhancement and Utilization, Nanjing Agricultural University, Nanjing 210095, China; 2022104054@stu.njau.edu.cn (N.Z.); 2023204050@stu.njau.edu.cn (C.C.); jiawenxiong2001@gmail.com (J.-W.X.);

**Keywords:** *Camellia sinensis*, chlorophyll metabolism, expression patterns, photoperiodic regulation, high-throughput transcriptomics

## Abstract

Tea plants are a perennial crop with significant economic value. Chlorophyll, a key factor in tea leaf color and photosynthetic efficiency, is affected by the photoperiod and usually exhibits diurnal and seasonal variations. In this study, high-throughput transcriptomic analysis was used to study the chlorophyll metabolism, under different photoperiods, of tea plants. We conducted a time-series sampling under a skeleton photoperiod (6L6D) and continuous light conditions (24 L), measuring the chlorophyll and carotenoid content at a photoperiod interval of 3 h (24 h). Transcriptome sequencing was performed at six time points across two light cycles, followed by bioinformatics analysis to identify and annotate the differentially expressed genes (DEGs) involved in chlorophyll metabolism. The results revealed distinct expression patterns of key genes in the chlorophyll biosynthetic pathway. The expression levels of *CHLE* (*magnesium-protoporphyrin IX monomethyl ester cyclase gene*), *CHLP* (*geranylgeranyl reductase gene*), *CLH* (*chlorophyllase gene*), and *POR* (*cytochrome P450 oxidoreductase gene*), encoding enzymes in chlorophyll synthesis, were increased under continuous light conditions (24 L). At 6L6D, the expression levels of *CHLP1.1*, *POR1.1,* and *POR1.2* showed an oscillating trend. The expression levels of *CHLP1.2* and *CLH1.1* showed the same trend, they both decreased under light treatment and increased under dark treatment. Our findings provide potential insights into the molecular basis of how photoperiods regulate chlorophyll metabolism in tea plants.

## 1. Introduction

Tea plants (*Camellia sinensis* (L.) O. Kuntze) are one of the most important cash crops in the world, and its leaves play an important role in tea production. The content and activity of chlorophyll in the leaves has a decisive impact on the growth of the tea plant and tea quality [1]. Chlorophyll is an indispensable pigment for photosynthesis in plants, which gives plants their characteristic green color and plays a crucial part in the conversion of light energy into chemical energy [2]. When a plant lacks chlorophyll, abnormal manifestations will appear, such as bleaching or yellowing. In addition, when the chlorophyll degradation process is blocked, excessive chlorophyll accumulation might result, which in turn produces reactive oxygen species, causing cell damaged and even death [3,4].

In higher plants, chlorophyll biosynthesis occurs, which is a finely regulated multi-stage process involving the action of many enzymes [5]. Chlorophyll *a* and chlorophyll *b* are indispensable pigments for plant photosynthesis, and their synthesis process is mainly divided into three key stages [6,7]. Until now, cloning and functional studies of related genes in model plants, such as *Arabidopsis thaliana,* have provided important information to understand the molecular mechanism of chlorophyll synthesis.

In tea plants, methods to increase chlorophyll content have mainly included shading, genetic engineering, and environmental control [8,9,10]. Under natural light and shade conditions, the upregulated expression of the *CPOX (coproporphyrinogen oxidase gene)* gene may lead to the increased accumulation of chlorophyll synthetic raw materials, while the downregulated expression of the *SGR (stay-green gene)* gene may lead to reduced chlorophyll degradation. These two changes jointly promote the increase in the total chlorophyll content in ‘Fuding white tea’ [8]. Ma et al. increased the chlorophyll content in tea plants through plastic greenhouse mulching cultivation, which made the tea greener in color and significantly increased the chlorophyll content, stomatal conductance, transpiration rate, and net photosynthetic rate. It also activated the gene expression and enzyme activity related to chlorophyll metabolism in tea plants [1].

The circadian clock is a biological mechanism that regulates physiological and biochemical activities in cycles of approximately 24 h, helping organisms adapt to periodic changes in the external environment, such as light and temperature changes [11,12]. In plants, the circadian clock is crucial for regulating many physiological processes, including the biosynthesis of chlorophyll, photosynthesis, and the synthesis and degradation of starch. Under normal light conditions, tea plants can carry out efficient photosynthesis and maintain an appropriate level of chlorophyll content. Changes in light intensity, such as under strong or weak light stress, can affect the balance between the synthesis and degradation of chlorophyll, thereby affecting the chlorophyll content. Studies have found that insufficient light does affect the biosynthesis of chlorophyll in plants, with most plants being unable to synthesize chlorophyll in the dark. Alternatively, excessively strong light can cause photoinhibition in plants, also inhibiting the synthesis of chlorophyll [13,14]. The photoperiod refers to the alternating changes in the light and dark periods within a daily cycle, involving the plant’s perception of different light conditions, distinguishing the length of daylight, and regulating the internal circadian clock responses. Ma et al. used grapevine rootstocks and variegated grape seedlings as experimental materials and studied the effects of different photoperiods on leaf pigment content. The results indicated that the shorter the photoperiod, the poorer the capacity for chlorophyll synthesis [15]. Until now, there has been little research on the content of chlorophyll in tea leaves and the related metabolic gene expression under different photoperiods.

As transcriptome sequencing technology becomes increasingly sophisticated, high-throughput sequencing has been applied in the field of tea plant research, becoming an important tool for tea plant genomics studies and breeding programs [16,17,18]. In this study, we conducted chlorophyll content and carotenoid content measurements for the tea plant ‘Baiye 1’ under two treatments: a skeleton photoperiod (6L6D) and continuous light conditions (24 L) at a photoperiod interval of 3 h (24 h). Additionally, transcriptome sequencing was performed at six different time points within two light cycles. Bioinformatics methods were used for the sequence analysis, functional annotation, functional classification, and metabolic pathway analysis. We also screened for differential genes involved in chlorophyll metabolism and confirmed the expression profiles of tea plant-related chlorophyll metabolism genes, using real-time fluorescent quantitative PCR technology. By analyzing the chlorophyll metabolism genes at different time points, we can reveal how the photoperiod regulates the synthesis and degradation of chlorophyll. The periodic expression patterns of chlorophyll metabolism genes can provide important information for studying the plant circadian clock, helping to understand how plants regulate physiological processes through their circadian clock.

## 2. Results

### 2.1. Chlorophyll and Carotenoid Content in Tea Plants Under Different Photoperiods

**Figure 1:**
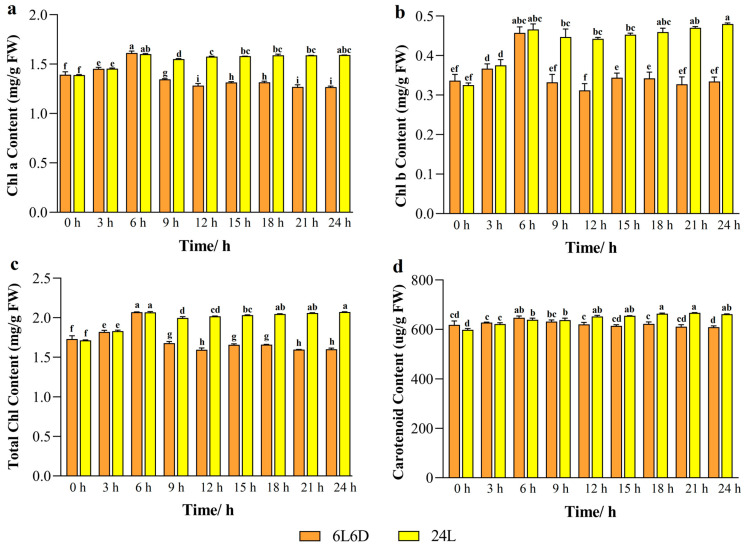
Changes in chlorophyll and carotenoid content in tea plants under two different photoperiods. (**a**): Chl *a* content; (**b**): Chl *b* content; (**c**): Total Chl content; (**d**): Carotenoid content. Orange represented skeleton photoperiod (6L6D), yellow represented continuous light conditions (24 L). The experimental material was ‘Baiye 1’ (seedling stage), a two-year-old tea plant cutting seedling. Statistical analysis was performed using IBM SPSS Statistics (version 25.0); the standard deviation (SD) is represented by the error bars. Duncan’s multiple comparison method was used to analyze the significance of the difference between the data at the 0.05 level (*p* < 0.05), different lowercase letters indicated significant difference at the 0.05 level (*p* < 0.05).

In higher plants, the photoperiod plays an important role in plant growth and pigment synthesis. The chlorophyll *a* content in tea leaves was higher than that of the skeleton photoperiod (6L6D) under continuous light conditions (24 L). This indicated that continuous light is conducive to the synthesis and accumulation of chlorophyll. The difference in Chl *a* content was the largest at 24 h of treatment, and the Chl *a* content was 1.25 times that of the skeleton photoperiod under long-day conditions, which might be related to the improvement in the photosynthesis efficiency. Under the 24 L conditions, the Chl *b* content reached the highest at 24 h of treatment, which was 0.48 mg/g. Under the skeleton photoperiod, the variation trend in the Chl *b* content showed that the synthesis tended to increase in the early light period (0 h–6 h), but decreased in the subsequent dark phase (6 h–12 h), which might be related to the decrease in photosynthetic activity in the dark phase. The trend in the carotenoid content was similar to that of the chlorophyll content, while under 24 L conditions, the carotenoid content showed an overall upward trend and reached the highest value at 21 h of treatment, which was 667.2 μg/g (Figure 1). According to the above results, tea plants perform photosynthesis and synthesize chlorophyll and carotenoids under light conditions (0 h–6 h). After the end of the light period, the tea plants returned to dark respiration (6 h–12 h) and the tea plants were unable to carry out photosynthesis to produce energy, chlorophyll synthesis was reduced at this time, while carotenoids accumulated due to the protective mechanisms designed to reduce the light inhibition during photosynthesis. Under secondary light conditions, the chlorophyll content was slowly upregulated, indicating that the tea plants may have adapted to specific photoperiods during evolution and, therefore, may not be able to effectively regulate chlorophyll and carotenoid synthesis during atypical photoperiods. 

### 2.2. Quality Analysis of Tea Transcriptome Sequencing at Different Time Points

Through transcriptome sequencing analysis of the tea leaf RNA at different time points (T0 h, T6 h, T12 h, T18 h, T24 h, F24 h) of the tea plant “Baiye 1”, 5.78~6.08 Gb of effective data were obtained at each time point. The value of Q20 (error rate less than 1%) is above 97.84% and the value of Q30 (error rate less than 1%) is above 94.42%. The larger the value, the higher the sequencing quality. The percentage of GC content was 44.054%, 43.882%, 43.767%, 44.213%, 44.225%, and 44.188%, respectively (Table 1).

Note: Sample is the name of the sample; clean reads are the number of remaining reads after filtration; clean bases are the number of bases remaining after clean base filtration; the Q20 rate is the proportion of bases with a mass value greater than 20 (error rate less than 1%) in the total sequence after filtration; the Q30 rate is the proportion of bases with a mass value greater than 30 (error rate less than 0.1%) in the total sequence after filtration; the GC content indicates the GC content of the filtered data.

### 2.3. Functional Annotation and Classification 

In order to reveal the biological function of the new unigenes, we performed an in-depth functional annotation analysis of 37,644 unigene sequences. We utilized the following seven databases: Nr (NCBI non-redundant), proposed by Deng [19], with a total of 27,713 transcripts matched in this database, accounting for 73.62%; Pfam (Protein family database), proposed by Finn [20], with 15,849 transcripts annotated in this database, accounting for 42.10%; 433 transcripts (72.87%) were annotated in Uniprot [21]; KEGG (Kyoto Encyclopedia of Genes and Genomes), proposed by Kanehisa, with 8050 transcripts annotated, accounting for 21.83% [22]; GO (Gene Ontology), proposed by Ashburner, with 20,207 transcripts annotated in this database, accounting for 53.68% [23]; and COG (Clusters of Orthologous Groups), proposed by Tatusov, with 98 transcripts annotated in this database, accounting for 0.26% [24]. Through this extensive database search, we were able to provide a wealth of functional information on these transcripts to better understand their biological roles (Table 2).

**Figure 2:**
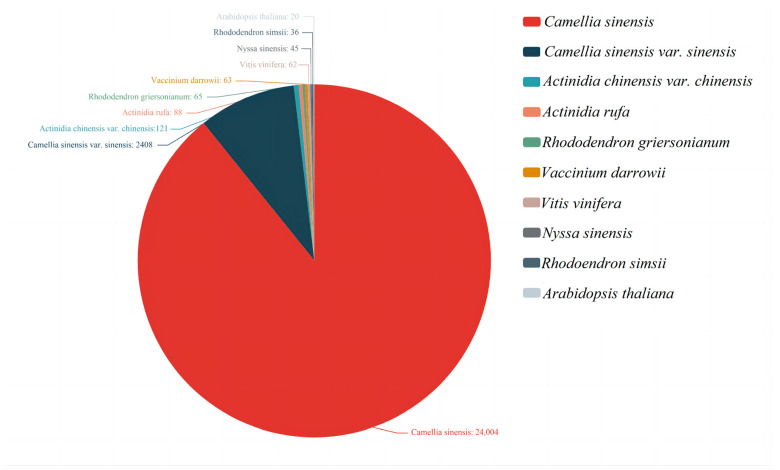
Nr annotated species distribution map for *C. sinensis*. The top 10 species with the most comparisons are counted. The number represents the number of different species in the Nr database. The different colors represent the different species. The species distribution of BLAST hits for each unigene occurs with a cut-off of 1E^−5^.

The assembled 37,644 unigenes were compared with the relevant databases and 27,713 unigenes were annotated in the Non-redundant Protein Database (Nr). According to the results of the Nr database comparison notes, the top 10 species with the most comparisons were counted and the rest were divided into other species. The species with the most comparisons were as follows: *Camellia sinensis* (24,044), *Camellia sinensis var. sinensis* (2408), *Aactinidia chinensis var. chinensis* (121), *Actinidia rufa* (88), *Rhododendron griersonianum* (65), *Vaccinium darrowii* (63), *Vitis vinifera* (62), *Nyssa sinensis* (45), *Rhododendron simsii* (36), and *Arabidopsis thaliana* (15) (Figure 2).

### 2.4. GO Analysis 

**Figure 3:**
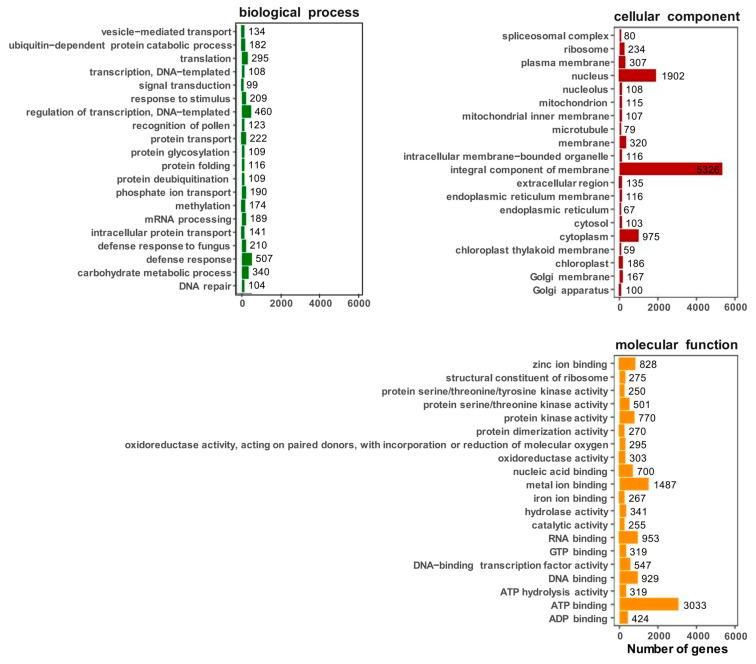
GO annotation of functional genes in *C. sinensis*. Select the top 20 most annotated GOslim under each classification; each unigene was classified into at least one GO term. All the unigenes were grouped into three categories: molecular function, cellular component, and biological process.

The expression of *C. sinensis* genes were searched in the GO database and the standardized gene functions were classified. A total of 20,207 unigenes were assigned to three main GO categories (biological process, cellular component, and molecular function) (Figure 3). In regard to the biological processes’ transcription regulation, DNA-templated synthesis (351), the defense response (273), and translation (translation, 241), involved the largest number of single-gene clusters. Among the cell components, integral components of the membrane (3857), the nucleus (1395), and cytoplasm (697), were involved in most single-gene clusters. The most common terms in regard to the molecular functions were ATP binding (ATP binding, 2173), metal-ion binding (1161), and RNA binding (RNA binding, 727).

### 2.5. KEGG Classification 

**Figure 4:**
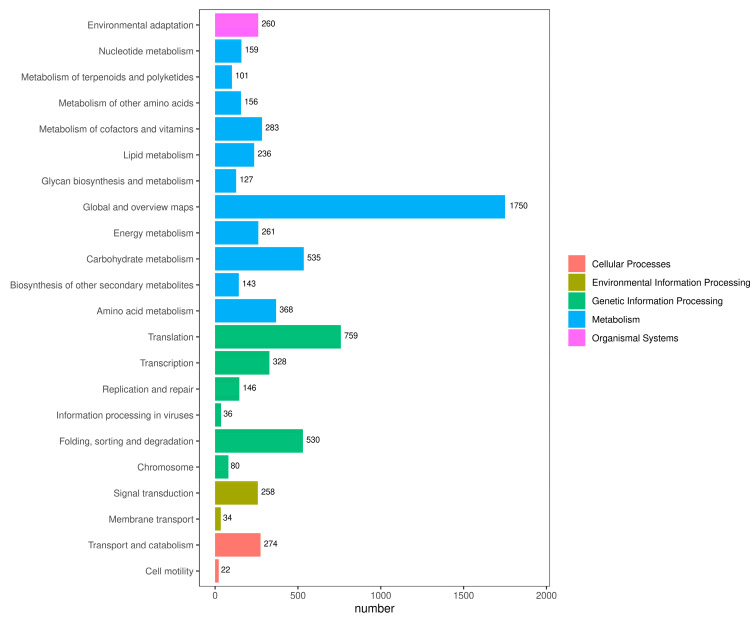
KEGG metabolism pathway categories in *C. sinensis.* Different colors represent the five classes involved in the KEGG metabolic pathway and the numbers represent the number of single genes in different classes.

Following the KEGG annotation of the unigene sequences, we grouped them based on the KEGG metabolic pathways in which they participated (Figure 4). A total of 8050 individual gene clusters were identified. The categorization was structured into five distinct tiers: cellular processes, environmental information processing, genetic information processing, metabolism, and organismal systems. Metabolism was the largest category, with 11 sublevels. The first three categories of these 11 sublevel metabolic pathways were global and overview maps (1750), carbohydrate metabolism (535), and amino acid metabolism (368). There were four sublevels of genetic information processing, with translation (759) and folding, sorting, and degradation (530) accounting for the majority.

### 2.6. KOG Classification

**Figure 5:**
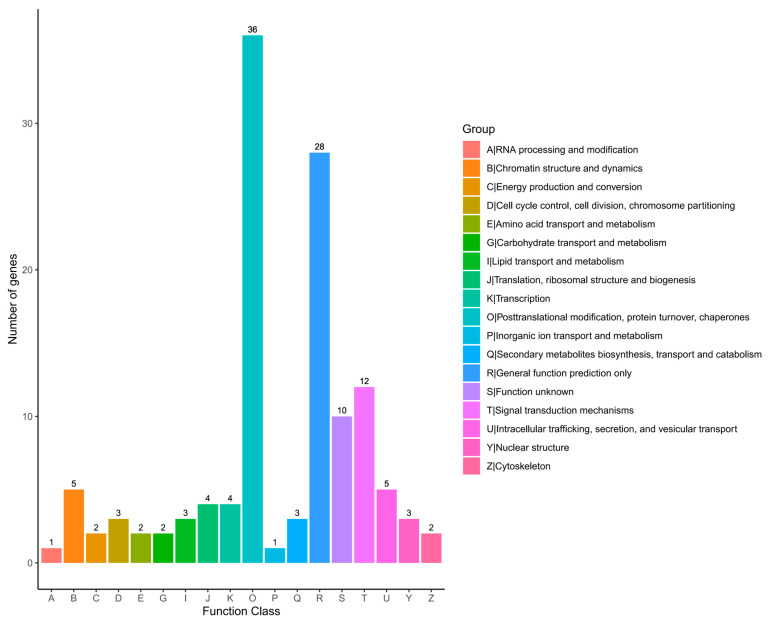
Clusters of orthologous group classifications.

The KOG database is a phylogenetic relational database specially constructed for eukaryotes. The KOG database divides eukaryotic genes into 18 different functional categories, which cover everything from basic biological processes to complex cellular functions. Through KOG annotating the unigenes, we were able to classify them according to the KOG functional group. In the results shown in Figure 5, a total of 98 sequences were classified in the KOG database. Of the 18 KOG classes, 10 (10.20%) of the single gene functions were not clear. Among the categories with known functions, “Posttranslational modification, protein turnover, chaperones” (post-translational modification, protein degradation, chaperones) was the largest category, containing 36 single genes, accounting for 36.73% of the total. The importance of this category lies in the fact that it involves the ultimate maturation, stability, and functional regulation of proteins, which are all critical steps for proteins within cells in order to function properly. This was followed by the “General function prediction only” category, which contained 28 single genes, accounting for 28.57% of the total. This category may include some genes that have a wide range of functions or are not yet well understood.

### 2.7. Screening of Differentially Expressed Genes in the Tea Transcriptome at Different Time Points

**Figure 6:**
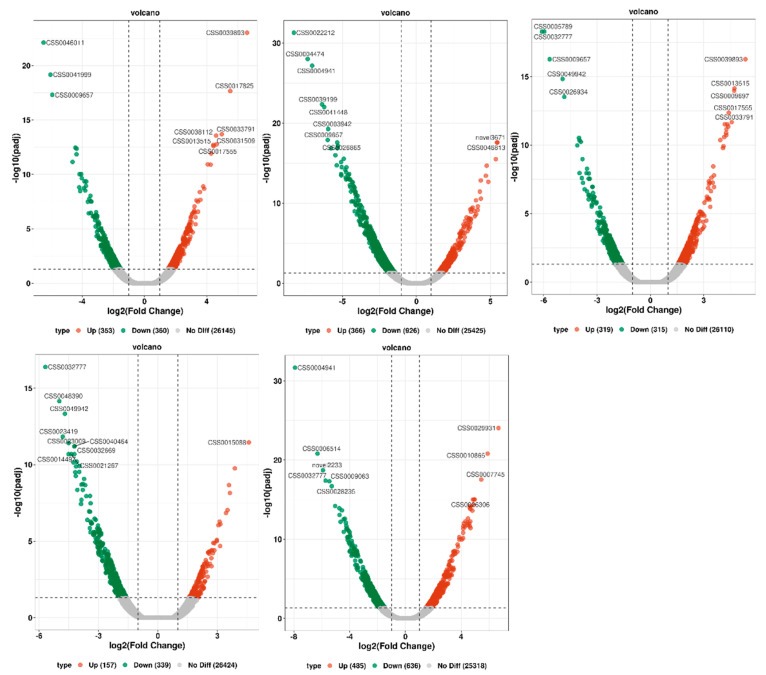
Volcano map of differentially expressed genes. Each dot on the plot signifies a gene, with the x-axis showing the change in gene expression, measured as the log2 fold change. The y-axis shows the significance of these changes, presented as the negative log10 of the *p*-value or the adjusted FDR.

Tea leaves at five different time points (T6 h, T12 h, T18 h, T24 h, F24 h) were compared with the initial time T0 h, and multiple differently expressed genes (DEGs) were identified, and the overall distribution of the different genes was inferred using a volcano map (Figure 6). The results showed that a total of 713 differential genes were expressed in T0 h versus T6 h, with 353 genes upregulated and 360 genes downregulated. A total of 1292 differential genes were expressed in T0 h vs. T12 h, with 366 genes upregulated and 926 genes downregulated. A total of 634 differential genes were expressed in T0 h versus T18 h, with 319 genes upregulated and 315 genes downregulated. A total of 496 differential genes were expressed in T0 h versus T24 h, of which 157 genes were upregulated and 339 genes were downregulated. A total of 1121 differential genes were expressed in T0 h versus F24 h, with 485 genes upregulated and 636 genes downregulated (Table 3).

### 2.8. Screening of Different Genes in the Chlorophyll Metabolic Pathway in Tea Plants at Different Time Points

**Figure 7:**
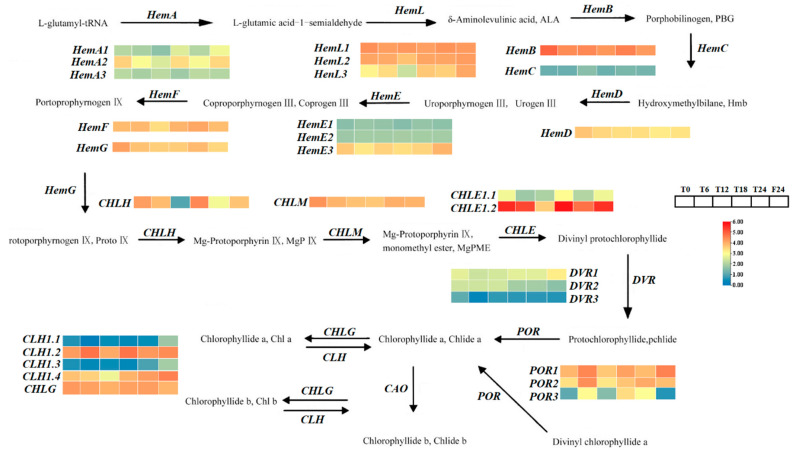
Differential expression of chlorophyll metabolic pathways and related genes [5]. Note: Red indicates upward adjustment, blue indicates downward adjustment. The heat map of the pathway shows the differential expression level of chlorophyll metabolism-related genes in tea leaves at 5 different time points (T6 h, T12 h, T18 h, T24 h, F24 h) and the initial time T0 h during 2 photoperiods. Blue and red are used to represent the difference multiples after log2, from low to high.

In the chlorophyll metabolism pathway of the tea plants at different time points, we screened nine differentially expressed genes involved in chlorophyll synthesis and degradation. In “T0 h vs. T6 h”, a total of three differentially expressed genes are upregulated, including one Geranylgeranyl reductase (*CHLP*), two cytochrome P450 oxidoreductase, and two cytochrome P450 oxidoreductase (*POR*) genes. There was also a downregulation of differentially expressed magnesium-protoporphyrin IX monomethyl ester cyclase (*CHLE*). In “T0 h vs. T12 h”, there were four differentially expressed downregulated genes, including one chlorophyllase gene (*CLH*), two *CHLE* genes, and one *CHLP* gene. In “T0 h vs. T18 h” and “T0 h vs. T24 h”, there was a total of two differentially expressed upregulated gene, both of which were *POR* genes. In “T0 h vs. F24 h”, there was a total of one differentially expressed upregulated gene, which was a *CLH* gene. Through the data obtained by transcriptome sequencing, heat maps were used to observe and analyze the expression level changes in the related genes for different time periods during chlorophyll metabolism, which may indicate the functions of these genes in the regulation of chlorophyll metabolism (Figure 7).

### 2.9. Fluorescence Quantitative PCR Verification

**Figure 8:**
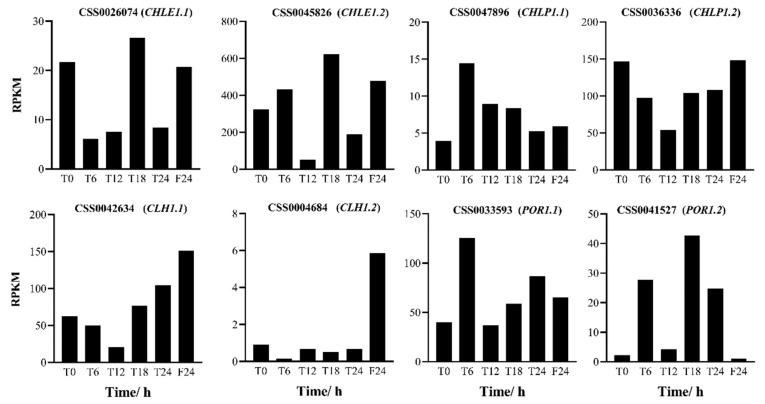
RPKM values of tea plants at different time points. T0 represents the initial time; T24 relates to 24 h in the skeleton photoperiod (6L6D); F24 relates to 24 h under constant light (24 L).

**Figure 9:**
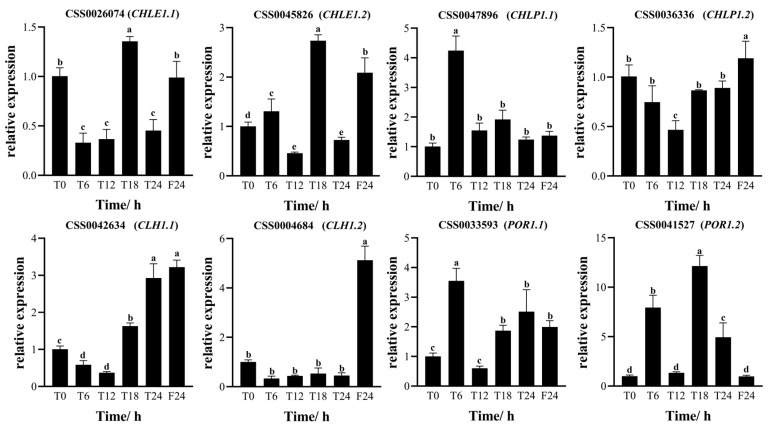
The expression levels of chlorophyll metabolic genes at different time points. T0 represents the initial time; T24 relates to 24 h in the skeleton photoperiod (6L6D); F24 relates to 24 h under constant light (24 L). Statistical analysis was performed using IBM SPSS Statistics (version 25.0); the standard deviation (SD) is represented by the error bars. Duncan’s multiple comparison method was used and different lowercase letters indicated significant difference at the 0.05 level (*p* < 0.05).

Eight differential genes in the chlorophyll metabolic pathway were analyzed by RT-qPCR. The results showed that the trends as seen in the quantitative fluorescent PCR were consistent with the transcriptome sequencing results (Figure 8 and Figure 9). The expression patterns of the *CHLE1.1* gene and *CHLE1.2* gene were essentially the same, both peaking at T18 and F24. Under the 6L6D photoperiod treatment, the expression levels of the *CHLE1.1* gene and *CHLE1.2* gene reached their peak at T18. The expression levels of the *CHLP1.1*, *POR1.1*, and *POR1.2* genes showed an oscillatory trend, characterized by an increase–decrease–increase pattern. The expression trends of the *CHLP1.2* gene and *CLH1.1* gene were basically consistent, decreasing during the day and increasing at night. The expression level of the *CLH1.2* gene was low throughout the day. Under continuous light (24 L), the expression levels of all the chlorophyll genes increased. Among them, the expression level of the *CHLE1.2* gene at F24 (after 24 h of treatment) was 2.09 times that at T0. The expression levels of the *CLH1.1* and *CLH1.2* genes at F24 were even higher, being 3.22 and 5.12 times that at T0, respectively.

## 3. Discussion

The photoperiod is one of the most important environmental factors in plant growth and development. Under artificial lighting conditions, the photoperiod can be precisely adjusted according to the specific needs of production, thus breaking nature’s inherent day–night alternating pattern. Sysoeva et al. pointed out that continuous light can significantly affect the flowering time and biomass accumulation of certain plants [25]. Relevant studies have also emphasized the significance of constant light in controlling plant growth and how it can greatly increase crop yields [26,27,28]. Li et al. increased the absorption and accumulation of nitrogen and phosphorus, especially potassium, in cucumber seedlings by extending the light time. The accumulation of micronutrients in cucumber seedlings also increased with the extension of light supplement time [29]. Our study found that under the condition of continuous light (24 L), the chlorophyll content and carotenoid content of tea plants were higher than that of the skeleton photoperiod (6L6D), indicating that continuous light is conducive of chlorophyll synthesis and accumulation. On the contrary, the Chl content of leafy vegetables (such as leaf lettuce, red amaranth, red spinach, Swiss chard, red chard, green amaranth, etc.) when the photocycle (light/dark) was 12 h/12 h was higher than that of 18 h/6 h and 24 h/0 h [30]. Park et al. cultured lettuce in an incubator with several photoperiods [12/12, 18/6, or 24/0 (light/dark)] and found that leaf length, width, fresh weight, dry weight, and the total anthocyanin content were all the highest during the 24/0 photoperiod, but the chlorophyll value was the highest during the 12/12 photoperiod, higher than the 18/6 and 24/0 photoperiod [31].

By shortening the growth cycle of plants, production efficiency can also be improved, and this strategy has important application value [32]. Although maintaining an optimum daily light duration is critical for plant growth, there has been little research on how modifying the light–dark cycle can promote plant growth. In this study, tea plants were placed in a skeleton photoperiod (6L6D) environment, and it was discovered that the chlorophyll content of tea plants increased in the early period of light (0–6 h), while chlorophyll synthesis was inhibited in dark conditions, which may be related to the insufficient light energy required in terms of the photosynthetic pathway. When the plant was exposed to light again, the chlorophyll content gradually recovered, indicating that the plant is adaptable. This adaptability may be linked to the regulation of the plant’s internal biological clock, which helps the plant adapt to periodic changes in the external environment by regulating the physiological and biochemical activities. The change pattern in carotenoid concentration was comparable to that of chlorophyll, while under long-day settings, the carotenoid content increased generally, which could be associated with the photoprotection mechanism [11,12]. He et al. investigated the effects of three different photoperiods on tomato plants and discovered that under short-day settings (7 h of light/5 h of darkness and 3.5 h of light/2.5 h of darkness), tomato leaves displayed indications of green deficit [33]. Kang et al. [34] further investigated the effects of light intensity and the photoperiod on the growth and morphology of lettuce, and the results showed that high light intensity (290 μmol·m^−2^·s^−1^ PPFD (Photosynthetic Photon Flux Density), photosynthetically active radiation density) combined with a short photoperiod (6 h light/2 h darkness) was beneficial to the overall growth and development of lettuce. Medium light intensity (230 or 260 μmol·m^−2^·s^−1^ PPFD) combined with a longer photoperiod (18 h light/6 h darkness and 9 h light/3 h darkness) was more conducive to plant growth and improved photosynthetic capacity. In addition, the physiological response of tea plants to the photoperiod, such as the diurnal change pattern in chlorophyll content, also reflects the plant’s adaptability to the photoperiod, which may be closely related to the regulatory mechanism of the plant’s internal biological clock.

The biological clock is a time regulation mechanism in living organisms that helps organisms adapt to periodic changes in the external environment. The chlorophyll requirements of plants vary with the change in light, from day to night [35]. The primary process in terms of plant growth and development is chlorophyll metabolism, which influences photosynthetic efficiency, and is essential for the plant’s response to environmental changes. Numerous enzymes and transcription factors govern the synthesis and degradation of chlorophyll in a fine-grained manner [36,37,38]. The circadian rhythm can regulate the expression of the genes encoding to the subunits of plant magnesium ion chelatase, but the regulation pattern is different. In *Arabidopsis thaliana*, the expression trends of *CHLI* (*Magnesium chelatase I*), *CHLD* (*Magnesium chelatase D*), and *CHLH* (*Magnesium chelatase H*) showed a similar circadian rhythm, and the expression levels of *CHLI*, *CHLD,* and *CHLH* reached their peak in darkness to light. In tobacco, the expression trends of *CHLH* and *CHLI* have the same rhythm, the expression regularity of *CHLD* is different, and the expression level reaches its peak during the transition from light to darkness [39]. The transcriptome analysis and RT-qPCR verification in this work demonstrated variations in the expression of important genes for chlorophyll synthesis under various photoperiods, offering fresh perspectives on how photoperiods affect chlorophyll metabolism. The entire biosynthetic pathway involves at least 15 enzymes encoded by 27 genes, and the activity of these enzymes directly affects the synthesis efficiency of chlorophyll and the photosynthetic capacity of plants [40,41].

The expression levels of the *CHLE1.2*, *CHLP1.1*, *POR1.1,* and *POR1.2* genes were significantly increased in both light treatment stages and decreased in the dark treatment stages. It is possible that under light conditions, plants need more chlorophyll to capture light energy and perform photosynthesis [42]. In *Arabidopsis thaliana*, the PORC protein levels were increased under high light conditions, while the greening capacity of the PORB protein under low light conditions was affected [43]. Deng et al. discovered that dark conditions dramatically inhibited peanut cell division, particularly the amount of PORA protein, which was strongly associated with chloroplast production, limiting chloroplast synthesis and chlorophyll accumulation. Under light conditions, 21 transcription factors had a significant impact on the transcription dynamics of several genes [44]. On the contrary, the expression levels of the *CHLE1.1*, *CHLP1.2*, *CLH1.1* and *CLH1.2* genes decreased under the first light treatment. The expression levels of *CHLE1.1* and *CLH1.2* increased under the first dark treatment. However, the expression levels of *CHLP1.2* and *CLH1.1* increased under the second dark treatment, which may be related to the physiological adjustment of plants to reduce energy consumption and prepare for dormancy during the dark stage [45]. CLH2 played a role in chlorophyll degradation, its subcellular localization indicated that it was not involved in chlorophyll degradation during *Arabidopsis* senescence, suggesting that different CLH enzymes might have different functions and mechanisms of action in plant responses to light and dark periods [46]. In addition, under continuous light conditions, the expression level of key genes in chlorophyll synthesis were significantly increased, which was closely related to the response of plants to light and normal photosynthesis. The molecular regulation of chlorophyll production is a complicated process that iwas influenced by external environmental circumstances, growth and development, and the internal regulation of associated genes. POR catalyzes the formation of protochlorophyllide into chlorophylls under light conditions and is a light-dependent step in chlorophyll synthesis, which is critical for photosynthesis [47,48].

Other components of the circadian rhythm are also involved in the regulation of chlorophyll synthesis by light signals. In the darkness, *RVE1* (*REVEILLE1*) directly binds to the promoter of *PORA* to activate its expression. The expression level of *PORA* was significantly upregulated in *Arabidopsis* plants overexpressing *RVE1*, resulting in a decrease in Pchlide accumulation [49]. Hu et al. explored how circadian rhythms affect the expression of MYB transcription factor genes in tea plants, which are closely associated with chlorophyll metabolism [18]. With the deepening of research, mathematical models of plant circadian rhythms have been proposed, one after another. Huang et al. used tomato as a material to study how different light intensity affected the plant’s biological clock and photosynthesis [50]. Hu et al. found that under the photoperiod of the skeleton conditions (light/dark light = 6 h/6 h, 6L6D; light/darkness = 3 h/3 h, 3L3D), the expression profiles of the biological clock genes and photosynthesis-related genes of the tea plants were changed, and the circadian rhythm of the tea plant was more disturbed, the photosynthetic efficiency decreased, and the stomatal opening was irregular than that under normal light, but it still had a rhythm [51]. Plants’ biological clock senses photoperiod variations and regulates the expression of the photoperiod response genes to synchronize plant growth and development. However, the regulatory systems governing chlorophyll metabolism are more complex. In addition to photoperiodic impacts, environmental factors like temperature, water, and nutritional status may affect chlorophyll metabolism by modulating gene expression [52,53]. 

In this study, two atypical light cycles (24 L, 6L6D) were used to simulate different sunshine patterns to explore how the biological clock of tea plants responds to the challenges of these unnatural light conditions. Proper photoperiod management can also reduce unnecessary energy consumption. The impact of climate change on agricultural production is becoming more significant, thus understanding the impact of photoperiods on crop metabolism can help growers adopt adaptive management strategies to cope with unstable light conditions. We plan to continue this experiment by extending the days to observe cyclical changes in chlorophyll and carotenoid metabolism. Optimizing chlorophyll metabolism by regulating the photoperiod may help improve the resistance of tea plants to adversities, such as drought, low temperatures, etc. The results provide a scientific basis for cultivation management and the breeding of tea plants.

## 4. Materials and Methods

### 4.1. Plant Materials

The cutting seedlings of two-year-old ‘Baiye 1’ plants were cultivated in the State Key Laboratory of Crop Genetics and Germplasm Enhancement and Utilization, Nanjing Agricultural University (Nanjing, China, 188.84° E, 32.04° N). Tea seedlings with healthy growth were selected for growth in the light incubator (temperature 25 °C, photocycle 12 h/12 h, light intensity 240 μmol·m^−2^·s^−1^, humidity 70 ± 5%).

The seedlings were planted in loose, fertile, and well-aerated, slightly acidic soil (with organic matter content above 1–2% and a pH value of 6.0). After one week of cultivation, the tea seedlings were placed in two different photoperiod growth chambers, namely constant light (24 h light, 24 L) and the skeleton photoperiod (6 h light/6 h dark, 6L6D), with a temperature of 25 °C, a light intensity of 240 μmol·m^−2^·s^−1^, and humidity of 70 ± 5%. Both treatments began sampling at 9 am, with the initial time recorded as 0 h (Table 4). Healthy seedlings were selected, and one bud with two leaves was harvested every 3 h, then wrapped in aluminum foil, quickly frozen with liquid nitrogen, and stored at − 80°C for subsequent experiments. Samples were taken for transcriptome analysis from the skeleton photoperiod (6L6D) at ZT0, ZT6, ZT12, ZT18, ZT24, and constant light (24 L) at ZT24. Each sample was performed with three biological replicates.

### 4.2. Extraction and Measurement of Chlorophyll and Carotenoid Content

Choose tea leaves that are healthy and in good growth condition, then remove the veins and cut into pieces. Using a precision balance, weigh 0.10 grams of cut blades. Place the weighed leaves in a mortar, mix in a little of quartz sand and calcium carbonate powder to help break down the cell walls, then add a small amount of 95% ethanol and grind vigorously until the leaves are white. The extraction solvent was a mixture of acetone, anhydrous ethanol, and water (4.5:4.5:1.0), and the crushed leaves were mixed with the extraction solution. The extracts’ absorbance values at 470 nm, 649 nm, and 665 nm were calculated using SpectraMax id5 (Sunnyvale, CA, USA), which correspond to the absorption peaks of chlorophyll *a*, chlorophyll *b,* and carotenoids. The chlorophyll and carotenoid content were then calculated.

### 4.3. RNA Extraction, cDNA Library Construction and Sequencing

The extraction of the total RNA from tea leaves involved the use of an RNA extraction kit (RNA simple total RNA Kit, Tiangen Company, Beijing, China). The concentration of the RNA samples was determined using a micro ultraviolet detector NanoDrop ND-1000 spectrometer (spectrometer, Shanghai, China). The quality of the RNA was detected by 1.2% agarose gel electrophoresis. After that, six groups of RNA samples were sent to Bena Technology Co., Ltd. (Wuhan, China) for cDNA library construction and sequencing. The mRNA was extracted using the oligo (dT) magnetic bead technique. Break the mRNA into fragments suitable for sequencing. The interrupted mRNA was synthesized into single-stranded and double-stranded cDNAs, successively. The library was prepared by terminal repair, A-tail addition, sequencing joint connection, cDNA purification, and PCR amplification. Finally, high-throughput sequencing was performed using the DNBSEQ platform from BGI (Bena Technology Co., Ltd., Wuhan, China).

### 4.4. RNA-Seq Data and Enrichment Analysis of Differentially Expressed Genes 

The accuracy of the analysis results, while examining transcriptome sequencing data, is directly impacted by the quality of the original data. To guarantee high-quality reads, rigorous quality control and initial sequencing data pre-processing are therefore necessary. A quality assessment was performed on the raw sequencing data, using FastQC software (version 0.11.9, with default parameters, https://www.bioinformatics.babraham.ac.uk/projects/fastqc/, accessed on 15 July 2024)) to identify and exclude low-quality sequences and possible contaminants [54]. The Trim Galore tool was used to trim the sequences of the transcriptome samples, remove low-quality areas, and retain high-quality reads [55]. The processed reads were compared with the tea plant reference genome, using HISAT2 software (version 2.1.0) to determine the position of the reads on the genome [56]. The reads for each gene were counted, and RSEM software (version 1.3.3) was used (with default parameters) to estimate the number of reads per transcript. The read numbers obtained by RSEM were converted into FPKM values, a standardized expression measure used to assess gene and transcript expression levels [57,58]. The edgeR software (version 4.2.1) package was used to identify differentially expressed genes (DEGs) with a false discovery rate (FDR) threshold of less than 0.05 and a logarithm multiple change (logFC) threshold greater than 1.5. If the number of transcripts meeting these requirements is insufficient, the screening criteria should be further relaxed to a value of *p* < 0.05 and the absolute value of |logFC| < 1.5 [59]. In this analysis, the FDR values were used as the filtering criteria for significance differences to ensure that the differentially expressed genes identified were statistically significant.

### 4.5. Functional Annotation Analysis

The gene function of the tea plant ‘Baiye 1’ was annotated by sequence comparison with public databases. The BLAST tool was used for comparison in the NCBI’s Nr and Nt databases, Swiss-Prot and KOG databases, and the matching results with an e value of less than 1e^−5^ were screened. Blast2GO software (version 6.0) maps sequence functions to a gene ontology (GO) covering molecular functions, biological processes, and cellular components. The KEGG database is used for channel allocation. The coding region is identified, and the sequence direction is determined by the best matching method.

### 4.6. RT-qPCR Validation of Differentially Expressed Genes

In this study, real-time fluorescence quantitative PCR (RT-qPCR) was used to analyze the expression of the genes related to chlorophyll metabolism in tea plants. The detection primers were designed using the Primer Premier 6.0 software (version 6.0). Bio-Rad IQ5 fluorescence quantitative PCR platform was used with an SYBR Premix Ex Taq kit (TaKaRa, Dalian, China). The total RNA extracted from tea was reverse transcribed into cDNA using a reverse transcription kit (TaKaRa Biotech Co., Ltd., Dalian, China). The *CsGAPDH* gene was used as an internal reference, and the CSGAPDH-F and CSGAPDH-R primers were used for RT-qPCR analysis [60]. Samples were taken every 6 h for cDNA preparation over a certain photoperiod (24 h). The amplification system consisted of 20 µL: 10 µL SYBR Green I mix, 0.4 µL forward and reverse fluorescence quantitative primers, 2.0 µL cDNA, and 7.2 µL ddH_2_O [61]. The amplification procedure was set for denaturation at 95 °C for 5 min, denaturation at 95 °C for 10 s, annealing at 54 °C for 30 s, and extension at 65 °C for 15 s, for a total of 40 cycles. The final concentration of the primers in the reaction mixture was 0.2 µM. Three biological replicates were performed, and 2^−∆∆CT^ scans were used to calculate the relative gene expression levels [62]. The RT-qPCR primers of the chlorophyll metabolism genes and intrinsic reference genes are listed in Table 5. 

### 4.7. Data Processing and Analysis

Microsoft Excel 2019 was used to organize and sort the data. IBM SPSS statistical software (version 25.0)was used to evaluate the differences between the data and determine their statistical significance. GraphPad Prism 9.4 software (version 9.4) was used to create the charts to visually display the results.

## 5. Conclusions

In this study, high-throughput transcriptomics techniques were used to analyze the chlorophyll metabolism responses of tea plants to photoperiodic changes. The results showed that light conditions significantly affected the change in the daily rhythm content of chlorophyll, and the expression difference in key synthase genes in light and dark phases revealed the fine regulation of the photoperiod on plant metabolism. In particular, the upregulation of gene expression related to chlorophyll synthesis under a continuous light environment provides a new perspective for improving tea quality through photoperiod regulation. In general, this study has enriched the understanding of the chlorophyll metabolism rhythm of tea plants and provides a scientific basis for the accurate management of tea planting, which is conducive to promoting the high-quality development of the tea industry. In addition, the differentially expressed genes (DEGs) screened in this study and their RT-qPCR validation have deepened the understanding of the molecular mechanism of tea plant adaptation to light changes and have verified the accuracy of the transcriptome data. 

## Figures and Tables

**Table 1:** Statistics on the tea transcriptome data at six time points.

Sample	Clean Reads	Clean Bases	Q20 Rate	Q30 Rate	GC Content
T0 h	43,291,008	6,042,417,256	98.606%	95.866%	44.054%
T6 h	43,294,090	6,071,741,360	98.397%	96.011%	43.882%
T12 h	43,299,782	6,084,730,076	98.533%	96.154%	43.767%
T18 h	43,297,578	6,037,877,452	97.840%	94.419%	44.213%
T24 h	43,315,956	5,785,374,886	98.800%	96.873%	44.225%
F24 h	43,325,480	6,027,509,621	98.460%	96.353%	44.188%

**Table 2:** Statistics on the functional annotation results.

Item	Count	Percentage
All	37,644	100.00%
KEGG	8050	21.38%
Pathway	4156	11.04%
Nr	27,713	73.62%
Uniprot	27,433	72.87%
GO	20,207	53.68%
KOG (EuKaryotic Orthologous Groups)	98	0.26%
Pfam	15,849	42.10%
TF (Transcription factor)	758	2.01%

**Table 3:** Number of differentially expressed genes.

Comparison	Significant Diff Number	Up	Down
T0 h vs. T6 h	713	353	360
T0 h vs. T12 h	1292	366	926
T0 h vs. T18 h	634	319	315
T0 h vs. T24 h	496	157	339
T0 h vs. F24 h	1121	485	636

**Table 4:** Different photoperiod treatment test schemes.

	Time	ZT 0-ZT 3	ZT 3-ZT 6	ZT 6-ZT 9	ZT 9-ZT 12	ZT 12-ZT 15	ZT 15-ZT 18	ZT 18-ZT 21	ZT 21-ZT 24
Groups									
6L6D		6 h (09:00~15:00)		6 h (15:00~21:00)		6 h (21:00~ ^+ 1^03:00)		6 h (^+ 1^03:00~ ^+ 1^09:00)	
24 L		24 h (09:00~ ^+ 1^09:00)							

Note: ‘ZTxx’ denotes the processing time, where ZT0 and ZT24 represent the beginning and end of a day, respectively. The white areas in the table indicate light processing and the gray-shaded areas indicate dark processing. The ‘xx h (xx:xx~xx:xx)’ in the table represents the total duration of the light (dark) processing phase and the corresponding processing time (Beijing time). The superscript ‘+ 1’ indicates the time in the following day.

**Table 5:** Primers for RT-qPCR.

Gene	Forward Primer Sequence (5′-3′)	Reverse Primer Sequence (5′-3′)
*CsCHLE1.1*	TGAAGGCGAATCCAGAGT	ATACCGAGAGGCAGAAGAA
*CsCHLE1.2*	TGCCTCTCGGTATATGTGA	TCTCTTGAACTCTGGATTCTC
*CsCHLP1.1*	CCTTCATCATCTCCGCTAA	GCTCCAATCACAACATCAA
*CsCHLP1.2*	CATAGACCGCCGAGTAAC	TGAGGAAGAGACCGTTGA
*CsCLH1.1*	CGAAGCGTAGATGACCAA	GTGTTACAGGAGCAATAGTAG
*CsCLH1.2*	TTCAATCCACACTGCCTAA	TCTTCATTCTCACCATCCAA
*CsPOR1.1*	GTCGGCTCAATTACAGGTA	CCACATCACTCGCTTCTT
*CsPOR1.2*	GTTGATGATGGAGGACTTGA	CGGATTCTTCTTCGGATACA
*GAPDH*	TTGGCATCGTTGAGGGTCT	CAGTGGGAACACGGAAAGC

## Data Availability

Data are available upon request.

## References

[1] Ma X.M., Liu J.X., Li H.Y., Wang W.Z., Liu L., Wang P.Q., Hu J.H., Zhang X.F., Qu F.F. (2024). Greenhouse covering cultivation promotes chlorophyll accumulation of tea plant (*Camellia sinensis*) by activating relevant gene expression and enzyme activity. BMC Plant Biol..

[2] Wu J.Q., Cheng J., Xu C.M., Qi S.L., Sun W.R., Wu S. (2020). *AUREA* maintains the balance between chlorophyll synthesis and adventitious root formation in tomato. Hortic. Res..

[3] Hörtensteiner S. (2013). Update on the biochemistry of chlorophyll breakdown. Plant Mol. Biol..

[4] Geng R.D., Pang X.Q., Li X., Shi S.S., Hedtke B., Grimm B., Bock R., Huang J.R., Zhou W.B. (2023). PROGRAMMED CELL DEATH8 interacts with tetrapyrrole biosynthesis enzymes and ClpC1 to maintain homeostasis of tetrapyrrole metabolites in *Arabidopsis*. New Phytol..

[5] Beale S.I. (2005). Green genes gleaned. Trends Plant Sci..

[6] Suzuki J.Y., Bollivar D.W., Bauer C.E. (1997). Genetic analysis of chlorophyll biosynthesis. Annu. Rev. Genet..

[7] Cornah J.E., Terry M.J., Smith A.G. (2003). Green or red: What stops the traffic in the tetrapyrrole pathway?. Trends Plant Sci..

[8] Li G.Z., Chen X., Zhao Y.C., Zhao D.G. (2024). Gene expression regulation of the effect of shading on chlorophyll content in Fuding White Tea (*Camellia sinensis* L.). Tree Physiol..

[9] Li Y.C., Jeyaraj A., Yu H.P., Wang Y., Ma Q.P., Chen X., Sun H.W., Zhang H., Ding Z.T., Li X.H. (2020). Metabolic Regulation Profiling of Carbon and Nitrogen in Tea Plants [*Camellia sinensis* (L.) O. Kuntze] in Response to Shading. J. Agric. Food. Chem..

[10] Tamilselvi E., Anburaj J., Haripriya D., Santhosh A., Rajakumar G., Kavya P., Li X.H. (2023). Influence of shading intensity on chlorophyll, carotenoid and metabolites biosynthesis to improve the quality of green tea: A review. Energy Nexus.

[11] Koussevitzky S., Nott A., Mockler T.C., Hong F.X., Sachetto-Martins G., Surpin M., Lim J., Mittler R., Chory J. (2007). Signals from chloroplasts converge to regulate nuclear gene expression. Science.

[12] Sanchez S.E., Kay S.A. (2016). The Plant Circadian Clock: From a Simple Timekeeper to a Complex Developmental Manager. Cold Spring Harb. Perspect. Biol..

[13] Duanmu D.Q., Casero D., Dent R.M., Gallaher S., Yang W.Q., Rockwell N.C., Martin S.S., Pellegrini M., Niyogi K.K., Merchant S.S. (2013). Retrograde bilin signaling enables Chlamydomonas greening and phototrophic survival. Proc. Natl. Acad. Sci. USA.

[14] He J.J., Yang J.P., Yang H., Zhao X., Ye X.Y. (2014). Effects of light intensity and nitrogen supply on the dynamic characteristics of leaf SPAD value of rice canopy. J. Zhejiang Univ. (Agric. Life Sci.).

[15] Ma S.Y., Li S., Niu J.Y., Zhang Z., Liu Y., Xue C. (2010). Effects of different LED light on physiological and biochemical characters of grape rootstock plantlets. J. Gansu Agric. Univ..

[16] Wu Z.J., Li X.H., Liu Z.W., Xu Z.S., Zhuang J. (2014). De novo assembly and transcriptome characterization: Novel insights into catechins biosynthesis in *Camellia sinensis*. BMC Plant Biol..

[17] Liu Z.W., Li H., Liu J.X., Wang Y., Zhuang J. (2020). Integrative transcriptome, proteome, and microRNA analysis reveals the effects of nitrogen sufficiency and deficiency conditions on theanine metabolism in the tea plant (*Camellia sinensis*). Hortic. Res..

[18] Hu Z.H., Zhang N., Qin Z.Y., Li J.W., Yang N., Chen Y., Kong J.Y., Luo W., Xiong A.S., Zhuang J. (2024). Differential Response of MYB Transcription Factor Gene Transcripts to Circadian Rhythm in Tea Plants (*Camellia sinensis*). Int. J. Mol. Sci..

[19] Deng Y.Y., Li J.Q., Wu S.F., Zhu Y.P. (2006). Integrated nr database in protein annotation system and its localization. Comput. Eng..

[20] Finn R.D., Bateman A., Clements J., Coggill P., Eberhardt R.Y., Eddy S.R., Heger A., Hetherington K., Holm L., Mistry J. (2014). Pfam: The protein families database. Nucleic Acids Res..

[21] Apweiler R., Bairoch A., Wu C.H., Barker W.C., Boeckmann B., Ferro S., Gasteiger E., Huang H., Lopez R., Magrane M. (2004). UniProt: The Universal Protein knowledgebase. Nucleic Acids Res..

[22] Kanehisa M., Goto S., Kawashima S., Okuno Y., Hattori M. (2004). The KEGG resource for deciphering the genome. Nucleic Acids Res..

[23] Ashburner M., Ball C.A., Blake J.A., Botstein D., Butler H., Cherry J.M., Davis A.P., Dolinski K., Dwight S.S., Eppig J.T. (2000). Gene ontology: Tool for the unification of biology. The Gene Ontology Consortium. Nat. Genet..

[24] Tatusov R.L., Fedorova N.D., Jackson J.D., Jacobs A.R., Kiryutin B., Koonin E.V., Krylov D.M., Mazumder R., Mekhedov S.L., Nikolskaya A.N. (2003). The COG database: An updated version includes eukaryotes. BMC Bioinform..

[25] Sysoeva M.I., Markovskaya E.F., Shibaeva T. (2010). Plants under continuous light: Review. Plant Stress.

[26] Velez-Ramirez A.I., van Ieperen W., Vreugdenhil D., Millenaar F.F. (2011). Plants under continuous light. Trends Plant Sci..

[27] Ohyama K., Omura Y., Kozai T. (2005). Effects of air temperature regimes on physiological disorders and floral development of tomato seedlings grown under continuous light. HortScience.

[28] Demers D.A., Dorais M., Wien C.H., Gosselin A. (1998). Effects of supplemental light duration on greenhouse tomato (*Lycopersicon esculentum* Mill.) plants and fruit yields. Sci. Hortic..

[29] Li H.Y., Liu H.H. (2013). Effects of Supplementary Illumination at Night on Hormones Content and Nutrient Absorption of Cucumber Seedlings. Chin. Agric. Sci. Bull..

[30] Ali M.B., Khandaker L., Oba S. (2009). Comparative study on functional components, antioxidant activity and color parameters of selected colored leafy vegetables as affected by photoperiods. J. Food Agric. Environ..

[31] Park J.E., Park Y., Jeong B.R., Seungjae H. (2013). Growth of lettuce in closed-type plant production system as affected by light intensity and photoperiod under influence of white LED light. Agric. Food Sci..

[32] Gaudreau L., Charbonneau J., Vézina L. (1994). Photoperiod and Photosynthetic Photon Flux Influence Growth and Quality of Greenhouse-grown Lettuce. HortScience.

[33] He W., Chen D.Y., Hu X.T., Wang X.X., Chen L.H., Zhang H.C., Yang Z.C. (2018). Effects of different photoperiods and photonflux ratios of red and blur LEDs on growth and development of tomato plants. Acta Agric. Borealioccidentalis Sin..

[34] Kang J.H., KrishnaKuma S., Atulba S.L.S., Jeong B.R., Hwang S.J. (2013). Light intensity and photoperiod influence the growth and development of hydroponically grown leaf lettuce in a closed-type plant factory system. Hortic. Environ. Biotechnol..

[35] Stenbaek A., Jensen P.E. (2010). Redox regulation of chlorophyll biosynthesis. Phytochemistry.

[36] Eckhardt U., Grimm B., Hörtensteiner S. (2004). Recent advances in chlorophyll biosynthesis and breakdown in higher plants. Plant Mol. Biol..

[37] Bollivar D.W. (2006). Recent advances in chlorophyll biosynthesis. Photosynth. Res..

[38] Hu X.Y., Gu T.Y., Khan I., Zada A., Jia T. (2021). Research Progress in the Interconversion, Turnover and Degradation of Chlorophyll. Cells.

[39] Papenbrock J., Mock H.P., Kruse E., Grimm B. (1999). Expression studies in tetrapyrrole biosynthesis: Inverse maxima of magnesium chelatase and ferro chelatase activity during cyclic photoperiods. Planta.

[40] Zhao Y.Q., Wang W.J., Zhan X.H., Zhang M.Y., Xiao Y., Hou X.R., Gao M., Xiao B., Gao Y.F. (2024). CsCHLI plays an important role in chlorophyll biosynthesis of tea plant (*Camellia sinensis*). Beverage Plant Res..

[41] Tanaka R., Tanaka A. (2007). Tetrapyrrole biosynthesis in higher plants. Annu. Rev. Plant Biol..

[42] Ji D., Li Q., Guo Y., An W., Manavski N., Meurer J., Chi W. (2022). NADP+ supply adjusts the synthesis of photosystem I in *Arabidopsis* chloroplasts. Plant Physiol..

[43] Masuda T., Fusada N., Oosawa N., Takamatsu K., Yamamoto Y.Y., Ohto M., Nakamura K., Goto K., Shibata D., Shirano Y. (2003). Functional analysis of isoforms of NADPH: Protochlorophyllide oxidoreductase (POR), PORB and PORC, in *Arabidopsis thaliana*. Plant Cell Physiol..

[44] Deng Q., Du P., Gangurde S.S., Hong Y., Xiao Y., Hu D., Li H., Lu Q., Li S., Liu H. (2024). ScRNA-seq reveals dark- and light-induced differentially expressed gene atlases of seedling leaves in *Arachis hypogaea* L.. Plant Biotechnol. J..

[45] Chen H., Wu W.Q., Du K., Ling A., Kang X.Y. (2024). The interplay of growth-regulating factor 5 and BZR1 in coregulating chlorophyll degradation in poplar. Plant Cell Environ..

[46] Hu X., Jia T., Hörtensteiner S., Tanaka A., Tanaka R. (2020). Subcellular localization of chlorophyllase2 reveals it is not involved in chlorophyll degradation during senescence in *Arabidopsis thaliana*. Plant Sci..

[47] Zhang S., Heyes D.J., Feng L., Sun W., Johannissen L.O., Liu H., Levy C.W., Li X., Yang J., Yu X. (2019). Structural basis for enzymatic photocatalysis in chlorophyll biosynthesis. Nature.

[48] Wang F., Yan J.R., Chen X.Y., Jiang C.H., Meng S.D., Liu Y.F., Xu T., Qi M.F., Li T.L. (2019). Light Regulation of Chlorophyll Biosynthesis in Plants. Acta Hortic. Sin..

[49] Xu X., Chi W., Sun X., Feng P., Guo H., Li J., Lin R., Lu C., Wang H., Leister D. (2016). Convergence of light and chloroplast signals for de-etiolation through ABI4-HY5 and COP1. Nat. Plants.

[50] Huang T., Liu H., Tao J.P., Zhang J.Q., Zhao T.M., Hou X.L., Xiong A.S., You X. (2023). Low light intensity elongates period and defers peak time of photosynthesis: A computational approach to circadian-clock-controlled photosynthesis in tomato. Hortic. Res..

[51] Hu Z.H., Huang T., Zhang N., Chen C., Yang K.X., Sun M.Z., Yang N., Cheng Y., Tao J.P., Liu H. (2024). Interference of skeleton photoperiod on circadian clock and photosynthetic efficiency of tea plant: In-depth analysis of mathematical model. Hortic. Res..

[52] Yu X.L., Hu S., He C., Zhou J.T., Qu F.F., Ai Z.Y., Chen Y.Q., Ni D.J. (2019). Chlorophyll Metabolism in Postharvest Tea (*Camellia sinensis* L.) Leaves: Variations in Color Values, Chlorophyll Derivatives, and Gene Expression Levels under Different Withering Treatments. J. Agric. Food Chem..

[53] Li X., Zhang W., Niu D., Liu X.M. (2024). Effects of abiotic stress on chlorophyll metabolism. Plant Sci..

[54] Davis E.M., Sun Y., Liu Y., Kolekar P., Shao Y., Szlachta K., Mulder H.L., Ren D., Rice S.V., Wang Z. (2021). SequencErr: Measuring and suppressing sequencer errors in next-generation sequencing data. Genome Biol..

[55] Brown J., Pirrung M., McCue L.A. (2017). FQC Dashboard: Integrates FastQC results into a web-based, interactive, and extensible FASTQ quality control tool. Bioinformatics.

[56] Si Y.M., Xing Y.Q., Cai L. (2016). Differential splicing event analysis of liver tumor-educated blood platelets RNA-seq data with Hisat2 and MISO. J. Inn. Mong. Univ. Sci. Technol..

[57] Liao Y., Smyth G.K., Shi W. (2013). The Subread aligner: Fast, accurate and scalable read mapping by seed-and-vote. Nucleic Acids Res..

[58] Li B., Dewey C.N. (2011). RSEM: Accurate transcript quantification from RNA-Seq data with or without a reference genome. BMC Bioinform..

[59] Robinson M.D., McCarthy D.J., Smyth G.K. (2010). edgeR: A Bioconductor package for differential expression analysis of digital gene expression data. Bioinformatics.

[60] Li H., Liu Z.W., Wu Z.J., Wang Y.X., Teng R.M., Zhuang J. (2018). Differentially expressed protein and gene analysis revealed the effects of temperature on changes in ascorbic acid metabolism in harvested tea leaves. Hortic. Res..

[61] Dragan A.I., Pavlovic R., McGivney J.B., Casas-Finet J.R., Bishop E.S., Strouse R.J., Schenerman M.A., Geddes C.D. (2012). SYBR Green I: Fluorescence properties and interaction with DNA. J. Fluoresc..

[62] Pfaffl M.W. (2001). A new mathematical model for relative quantification in real-time RT-PCR. Nucleic Acids Res..

